# The Effects of* Punica granatum* Flower Extract on Skin Injuries Induced by Burn in Rats

**DOI:** 10.1155/2017/3059745

**Published:** 2017-01-19

**Authors:** Ebrahim Nasiri, Seyed Jalal Hosseinimehr, Jafar Akbari, Mohammad Azadbakht, Soheil Azizi

**Affiliations:** ^1^Traditional and Complementary Medicine Research Center, Mazandaran University of Medical Sciences, Sari, Iran; ^2^Faculty of Allied Medical Sciences, Mazandaran University of Medical Sciences, Sari, Iran; ^3^Faculty of Pharmacy, Mazandaran University of Medical Sciences, Sari, Iran

## Abstract

*Background*. We compared the efficacy of* P. granatum *(P) flower extract with that of silver sulfadiazine (SSD) for treating thermal burn injuries in rats.* Methods*. Ten Wistar rats in each group were topically given base cream, normal saline, cream containing 1% SSD, or creams containing 5% or 10%* Punica granatum* flower extract. The treatments were administered once daily until complete wound healing was observed. The wound area and healing time were assessed. In addition, percentage wound contraction and histopathological characteristics such as neovascularization and collagen formation were determined. The tannin content in* P. granatum* extract was determined.* Results*. The decrease in the average size of wounds on day 15 of the treatment was higher in rats treated with creams containing* P. granatum* extract than in rats treated with cream containing SSD (2.8 ± 0.9 cm^2^ versus 8.4 ± 3.2 cm^2^). The wounds completely healed on day 25 of the treatment in rats treated with creams containing* P. granatum* flower extract compared with those in rats treated with the other agents.* Conclusion*. These results indicated that* P. granatum *flower extract promoted wound healing in rats and could be used for managing burn injuries.

## 1. Introduction

Burn injury is a public health problem worldwide, especially in undeveloped countries that lack adequate medical facilities, in terms of morbidity, long-term disability, and mortality [1, 2]. Infection is the key complication of burns and is responsible for various health problems [1]. Burns are associated with high healthcare costs, multiple operative procedures, prolonged rehabilitation periods, long-term disability, prolonged hospitalization, loss of body extremities, infection, and even death [3–7]. Exposure to hot water is one of the most frequent causes of burns [6]. Healing of burn wounds is a complex process, and appropriate wound healing is crucial for the restoration of disrupted anatomical continuity and disturbed functional status of the skin [7]. Wound healing involves three phases, namely, inflammation, tissue formation, and tissue remodeling [8]. Creams containing 1% silver sulfadiazine (SSD) are primarily used for treating burn injuries because of the antibacterial activity of SSD [1, 4, 9, 10]. However, SSD is associated with several disadvantages such as delayed wound healing, development of resistance, renal toxicity, leukopenia, and adverse reactions [4, 5, 10]. Therefore, it is important to find a more effective drug with fewer side effects for treating burn injuries.

The use of natural products for treating burns is an important part of health management and is a resourceful method for providing more effective and cheaper healthcare options [11–13].* Punica granatum *Linn. (Punicaceae), commonly known as pomegranate, is a small tree native to the Mediterranean region [14, 15]. This tree is called Golnar in Iranian traditional medicine. Several studies have examined the efficacy of herbal medicines for healing wounds [11, 13, 15]. The fruits and flowers of Golnar are used in home remedies for treating gastric pain, gastrointestinal infection, bleeding or gastralgia, dysentery, and hemorrhagia and wound healing [15, 16]. This herbal medicine has antitumor, antidiarrheal, antiulcer, antifungal, antioxidant, and hepatoprotective properties [14, 17]*. P. granatum* extracts have been used for treating diabetes mellitus and microbial infections [18, 19]. In addition, few studies have shown that the extracts of pomegranate flowers have beneficial effects on healing wounds. In the present study, we compared the efficacy of creams containing* P. granatum* flower extract with that of a cream containing SSD on healing burn wounds in rats.

## 2. Material and Methods

### 2.1. Preparation of* P. granatum* Flower Extract

*Plant Material*.* P. granatum* flowers were purchased from a local herbal drug market (Sari, Iran) in the summer of 2013. The identity of these flowers was confirmed by senior botanist Dr. Mohammad Azadbakht at the Mazandaran University of Medical Sciences, Iran (Herbarium number: 1003). The flowers were dried at room temperature and were powdered using a grinder. Aqueous ethanol (70%) was gradually added to the powdered flowers, and the mixture was kept at room temperature without exposure to direct light for 72 h. After filtration, the solution was concentrated in a rotary evaporator under reduced pressure. The temperature of the rotary evaporator was from 40 to 50°C. The hydroalcoholic extract of the flowers was then powdered in a freeze dryer. The yield of the dried extract was 26.8%.

### 2.2. HPLC Analysis of* P. granatum *Flower Extract

*HPLC*. The concentration of tannic acid in the* P. granatum *flower extract was analyzed by performing HPLC. HPLC was performed using HPLC Knauer Smartline system (Knauer Association, Germany) containing 1000 mL pump, solvent delivery system, sampler injector, and photodiode array detector (model DAD 2800; all purchased from Knauer Association) set at 280 nm and attached to ChromGate software (version 3.1.7). Analysis was performed using an ODS-C18 column (250 × 4 × 6 mm, 5 *μ*m particle size; NUCLEODUR, Düren, Germany) and a corresponding guard column. All the solvents were filtered and degassed before injecting them into the column. Mobile phase was methanol-water-phosphoric acid (50 : 50 : 0.01), and the flow rate of the mobile phase was 1.0 mL/min. All the measurements were performed at ambient temperature.

### 2.3. Determination of Tannin in* P. granatum* Using Spectrophotometry Method

Tannin contained in* P. granatum* flower extract was determined for spectrophotometry by using Folin-Denis reagent [20]. In this method, tannic acid reducts Folin-Denis reagent (phosphomolybdic-phosphotungstic reagent) in alkaline solution. Absorbance of the product was determined at 760 nm by using UV 6505 spectrophotometer (Jenway, UK). The calibration (standard) curve was prepared using several concentrations of tannic acid (0.1–1 mg/mL). Three solutions containing 1 mg/mL* P. granatum* flower extract were prepared, and their absorbance was read in the same way as that of the standard solution of tannic acid. The concentration of tannins in the* P. granatum* flower extract was calculated using the calibration curve. All the reagents used for spectrophotometry were obtained from Merck (Germany).

### 2.4. Preparation and Formulation of Creams Containing 5% and 10%* P. granatum *Flower Extract

*P. granatum* flower extract was mixed with liquid paraffin, stearyl alcohol, cetyl alcohol, and Span 80 at 70°C. The aqueous phase was prepared by adding Tween 80, propyl and methyl paraben, and glycerin in distilled water and by heating to 70°C. Next, the aqueous and oil phases were mixed and were homogenized at 500 rpm for 15 min. The cream was allowed to cool at room temperature during homogenization. All formulations were stored at 4°C, 25°C, and 40°C for two weeks, and their stability was evaluated.

### 2.5. Study Animals

The experimental protocol for animal studies was approved by the Ethical and Research Committee of the Mazandaran University of Medical Sciences (number 118-92). Male Wistar rats (*N* = 50; weight: 160–200 g; age: 8–10 weeks) were obtained from the Mazandaran University of Medical Sciences. They were housed under standard conditions at room temperature with a 12 h light/dark cycle and were provided with laboratory food and water ad libitum. The rats were anesthetized by injecting 50 mg/kg sodium thiopental intraperitoneally, and their backs were shaved and cleaned with 70% alcohol. An insulating box with a base of 2 × 5 cm was prepared and was used to induce baseline burns in rats under controlled conditions. The rats were placed in the box in a supine position, with their backs exposed to hot water at 90°C for 6 s [4]. This method allowed the burning of an exact diameter of the skin and resulted in a regular, rectangular second-degree burn injury (approximately 10% of total body surface) on the back of the rats. This device helps to create a controlled burn in the animal model. All the rats were immediately resuscitated by injecting 5 mL normal saline intraperitoneally [4, 6].

The rats with burn injuries were randomly divided into five groups containing 10 rats each. Group 1 was the control group in which the rats were only treated using normal saline without any topical treatment. Rats in group 2 were treated with base cream lacking any effective agent. Rats in group 3 were treated with a cream containing 1% SSD (Behvarzan Pharmaceutical Company, Iran). Rats in groups 4 and 5 were treated with creams containing 5% and 10%* P. granatum *flower extract, respectively. All the treatments were initiated 24 h after the burn injury. Wounds were dressed once daily. Before dressing, the wounds in all the groups were washed with normal saline and the wound was covered by a similar thin layer of the cream in each group. After 72 hours, all of the group wounds were kept open after dressing. To quantify the rate of wound healing, the area of the lesions was determined on days 1, 3, 7, 10, 14, 20, 25, 30, and 33 after the burn injury. Wound area was measured in square millimeter on each experimental day by a ruler and a photograph. Percentage wound contraction at each time point was determined using the following formula: percentage wound contraction = (initial wound area − current area)/initial wound area × 100. Measurements were made daily by the same examiner by using a ruler and by taking photographs [7, 21].

### 2.6. Histological Analysis

Skin tissue samples for histological analysis were obtained under anesthesia by making a small excision in the center region of the wound on days 8 and 21 after the burn injury. The tissue samples were fixed in 10% formalin and were embedded in paraffin. The paraffin-embedded tissue blocks were cut into 4 *μ*m thick sections and were stained with hematoxylin and eosin. Light microscopy was performed to assess pathological changes such as angiogenesis, granulation tissue (GT) formation, and reepithelialization in the wounds [4, 6, 7]. The results are expressed as an average of 10 microscopic fields. Histopathological analysis was performed by evaluating reepithelialization. Reepithelialization was determined by measuring the following parameters: thickness of the epidermal layer, thickness of granular cell layer, organization of squamous cells, and extent of keratin layer and orthokeratin. Complete healing was evaluated by determining the degree of scar formation, collagen organization, and innervation and formation of hair follicles at 3 weeks after the burn injury. A score of 0–3 was assigned to all the parameters evaluated: 0 = absent, 1 = mildly present, 2 = moderately present, and 3 = strongly present [7]. Collagen organization was also scored on levels 0–3 as follows:

0 = −, lagging down, disorganized, and poorly oriented collagen fibers
1 = +, horizontally oriented 10–20% collagen fibers
2 = ++, horizontally oriented 30–40% collagen fibers
3 = +++, well-formed and horizontally oriented collagen fibers

*Bacteriological Assessment*. Swabs were taken from the burn wound area before dressing change on the 4th and 8th days. The swabs were collected and transferred to the laboratory for testing. In the quantitative count study, 0.5 mL of normal saline was added to each of the samples. Each sample dilution was spread onto Blood Agar and MacConkey Agar and the plates were incubated at 37°C for 24 h. Diagnostic test for the colonies was applied with Novobiocin test by a blinded histopathologist.

### 2.7. Statistical Analysis

Wound sites were assessed daily. Macroscopic evaluation was performed daily by the direct observation of wounds during dressing. Inflamed tissue was characterized by the presence of edema, secretion, redness, dark secretion or pus, pain, and bleeding during wound dressing. Statistical analysis was performed using SPSS software and MS Excel. One-way analysis of variance was used for comparing quantitative variables among the groups. Scheffe post hoc multiple comparisons test was used to compare the means among the groups. The differences between the groups were considered significant at *P* < 0.05.

## 3. Results

**Figure 1:**
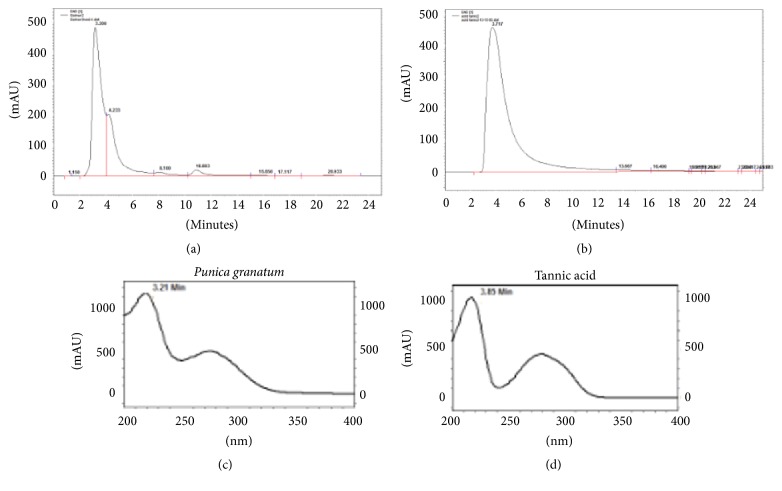
HPLC profile of* Punica granatum* total extract and tannic acid analyzed. (a) HPLC chromatogram of* Punica granatum* extract, (b) HPLC chromatogram of tannic acid (retention time: 3.8), (c) UV spectrum of peak* Punica granatum* extract with a retention time of 3.2, and (d) UV spectrum of tannic acid.

In this study, an isocratic elution of methanol in an acidic aqueous medium was used to analyze tannic acid concentration in the* P. granatum *flower extract. The separation of tannic acid is shown in Figure 1. Tannic acid standard has a typical retention time of 3.7 min, which was 3.8 min in the extract, and tannic acid in extract was confirmed with peak of pure tannic acid. The purity of the tannic acid peak in the HPLC chromatogram was confirmed using the photodiode array detector. The UV spectrum of pure tannic acid was the same as that of the tannic acid peak in the HPLC chromatogram of* P. granatum *flower extract (Figure 1). Tannin content in* P. granatum *flower extract was determined using a spectrophotometric method. Calibration curve from standard solution of tannic acid was prepared (*y* = 0.4593*x* + 0.0684, *R*^2^ = 0.9693) and with the help of this curve the tannin content of herbal extract was estimated. The mean weight of tannin present in* P. granatum* flower extract was 0.487 ± 0.035 mg/mg (*N* = 3) of the extract (48.71 ± 3.55 mg/100 mg of the extract, i.e., 48.7%).

**Figure 2:**
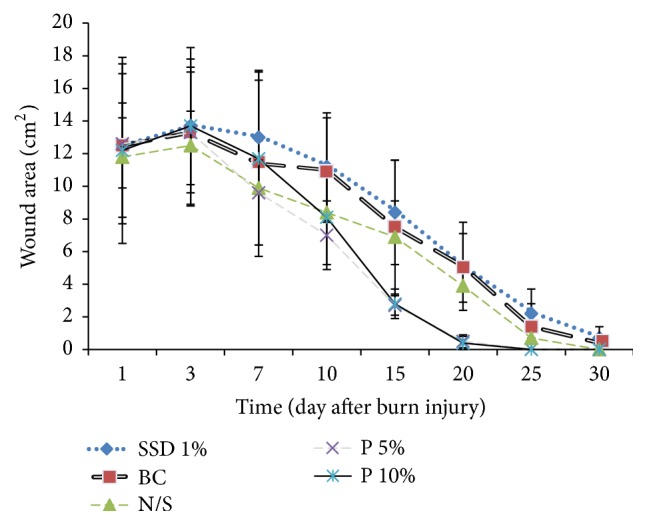
Mean wound area (cm^2^) of the animal groups treated with various topical creams. Silver sulfadiazine 1% (SSD 1%), normal saline (N/S), base cream (BC),* Punica granatum* flowers cream 5% (P 5%), and* Punica granatum* flowers cream 10% (P 10%). P 5% with SSD 1% (*P* < 0.003) and P 10% with SSD 1% (*P* < 0.004).

The effect of burn injury on weight loss in rats was recorded. No significant differences were observed among the groups with respect to weight loss induced by the burn injury. Skin lesions were measured on days 1, 3, 7, 10, 15, 20, 25, and 30 after the burn injury. The average wound sizes on the first day were 12.5 ± 2.6, 12.5 ± 4.4, 11.8 ± 2.4, 12.6 ± 4.9, and 12.2 ± 5.7 cm^2^ in rats treated with cream containing 1% SSD, base cream, normal saline, cream containing 5%* P. granatum* flower extract, and cream containing 10%* P. granatum* flower extract, respectively. Wound area in rats treated with creams containing* P. granatum* flower extract was lower by 1 cm^2^ on day 20 after the treatment (Figure 2).

Wound areas were not significantly different among the groups on days 3, 7, and 10 after the burn injury but were significantly different on day 15 after the burn injury (*P* < 0.001). Post hoc multiple comparisons Scheffe test showed that treatment of rats with creams containing 5%* Punica granatum* with 1% SSD was significant (*P* < 0.001) and 10%* P. granatum* flower extract showed smaller wound area on day 15 after the burn injury than rats treated with cream containing 1% SSD (*P* < 0.004).* The mean wound area of rats in the different groups is shown in Figure 2*. No significant difference was observed in wound size among rats treated with normal saline, base cream, and cream containing 1% SSD. However, a significant difference was observed in the wound size between rats treated with the base cream and creams containing 5% and 10%* P. granatum* flower extract (*P* < 0.024). The wound area was not different between rats treated with creams containing 5% and 10%* P. granatum* flower extract on day 15 after the burn injury.

The percentage wound contraction was significantly increased in rats treated with creams containing 5% and 10%* P. granatum* flower extract compared with that in rats treated with the other agents. Rats treated with cream containing 1% SSD showed longer healing time than those treated with creams containing* P. granatum* flower extract. These findings are summarized in Table 1.

Complete wound healing was observed on day 25 in rats treated with creams containing 5% and 10%* P. granatum* flower extract and on day 33 in rats treated with the other agents (Table 1).

**Figure 3:**
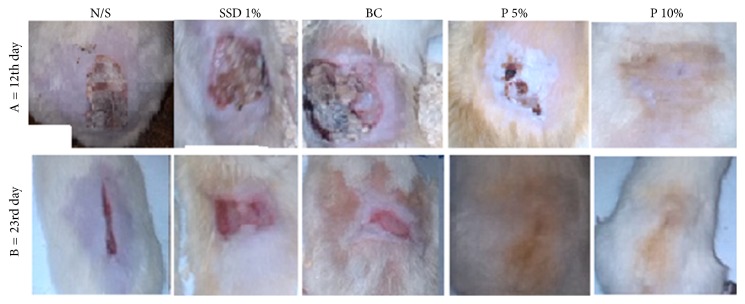
Comparison on dorsal wound condition between groups on the 12th day and 23rd day after burning. Silver sulfadiazine 1% (SSD 1%), normal saline (N/S), base cream (BC),* Punica granatum* flowers cream 5% (P 5%), and* Punica granatum* flowers cream 10% (P 10% in A = on the 12th day and B = on the 23rd day after burn injury).

These results indicated that wound healing in rats treated with* P. granatum* flower extract occurred 10 days before that in rats treated with the other agents. The condition of the burn wound on days 12 and 23 after the injury is shown in Figure 3.

Visual analysis of burn wounds treated with the base cream and cream containing 1% SSD showed redness and edema in the wound area; however, this was not observed in wounds treated with creams containing 5% and 10%* P. granatum* flower extract and with normal saline. Laboratory assessments showed no evidence of pathological bacteria. On days 5 and 8, a slight colorless secretion appeared in wounds treated with the base cream, 10%* P. granatum* flower extract, normal saline, and cream containing 1% SSD. Culturing of skin tissue samples treated with the above agents in Blood Agar yielded large, convex, round, and white colonies. Novobiocin test is used to differentiate coagulase-negative staphylococci. These bacteria were inferred to be* Staphylococcus epidermidis*, which are a part of the normal skin flora. Significant neovascularization and fibroblastic proliferation were observed on day 8 in rats treated with creams containing 5% and 10%* P. granatum* flower extract and 1% SSD compared with those treated with normal saline and base cream (Table 2).

GT formation was observed on day 8 in wounds treated with creams containing 5% and 10%* P. granatum* flower extract and 1% SSD compared with wounds treated with normal saline and base cream. GT formation score was higher in the 10%* P. granatum* flower extract group than in wounds treated with the other agents (Table 2).

**Figure 4:**
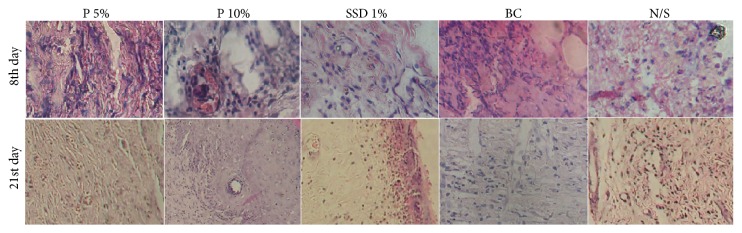
Comparison of the histopathology of biopsy samples from second-degree burns on 8 and 21 days after treatment with basic cream (BC), no treatment or normal saline (N/S), standard silver sulfadiazine treatment (SSD 1%), and* Punica granatum* 5% (P 5%) and* Punica granatum* 10% (P 10%) creams treatment. Neovascularization activity and fibroblastic proliferation were better in P 5%, P 10%, and SSD 1% cream compared with N/S and BC groups on the 8th day. Also, degree of granulation tissue was better in these groups. The thickness of the granular cell layer and the epidermal thickness degree in the wound of* Punica* 10% and SSD 1% wound treatment were better than in other groups on the 21st day.

On day 8, macrophage histiocytic infiltration and fibroblastic proliferation were similar in wounds treated with creams containing 5% and 10%* P. granatum* flower extract, base cream, and cream containing 1% SSD. Histopathological analysis of the dermis on day 21 after the burn injury showed that the degree of innervation and formation of lymphatic ducts in wounds treated with cream containing 10%* P. granatum* flower extract were better than those in wounds treated with the other agents. The results of histopathological analysis for reepithelialization showed that, on day 21, granular cell layer thickness and epidermal thickness in wounds treated with creams containing 10%* P. granatum* flower extract and 1% SSD were higher than those in wounds treated with other agents (Table 3). Furthermore, the formation of horizontally oriented collagen fibers of appropriate tension and strength in the scar tissue was better in wounds treated with creams containing 10% and 5%* P. granatum* flower extract than in those treated with the other agents (Figure 4).

## 4. Discussion

In this study, the efficacy of creams containing* P. granatum* flower extract was evaluated for treating burn wounds in rats. The results of this study showed that creams containing 5% and 10%* P. granatum* flower extract facilitated the healing of the burned tissue. Topical creams containing* P. granatum* flower extract were more effective in inducing wound healing than creams containing 1% SSD and other agents. On day 15 after the burn injury, the wound size was significantly smaller in rats treated with creams containing 5% and 10%* P. granatum* flower extract than in rats treated with the other agents.* P. granatum* has various pharmacological properties, including anti-inflammatory [22], antioxidant [23, 24], antibacterial [3], wound healing [25, 26], antifungal [14, 27], antispasmodic [28], and antiulcer properties [29, 30]. Our study showed that creams containing extracts of* P. granatum* flowers were more effective than creams containing 1% SSD for treating burn wounds. Treatment with creams containing the* P. granatum* flower extract accelerated the degree of GT formation compared to treatment with base cream and normal saline. The extent of scar tissue and hair follicle formation was higher in wounds treated with creams containing the* P. granatum* flower extract than in wounds treated with the other agents. The time for wound healing was shorter for wounds treated with creams containing* P. granatum* flower extract than for wounds treated with the other agents. Collagen organization was higher in wounds treated with creams containing* P. granatum* flower extract than in wounds treated with the other agents. Preliminary chemical analysis of the* P. granatum *flower extract showed that it contained high concentrations of tannin (0.487 mg/mg, 48.7%). Previous studies have reported that* P. granatum* extracts contain polyphenolic compounds such as ellagic acid, 3,3′,4′-tri-*O*-methyl ellagic acid, ethyl brevifolin carboxylate, maslinic acid, daucosterol, and tannins [31–33]. Singh et al. (2002) reported that* Punica* peel and seed extracts had antioxidant properties and suppressed peroxidation [24]. Tannins exert antibacterial effects against many bacteria. Tannins are polyphenolic compounds containing hydroxylic, carboxylic, and other hydrophilic groups and are considered to be macromolecules [31, 34, 35]. Although tannins are known to exert antibacterial effects and promote wound healing, the mechanisms underlying these effects are unclear [36, 37]. Our study has shown that* P. granatum* flower extract contains a high level of tannins. This effective material probably facilitates wound healing in this research study.

Wound healing is a multiphase process characterized by wound contraction, granulation, epithelialization, and collagenation. Wound healing involves 3 phases, that is, inflammation, proliferation, and remodeling [38, 39]. Proliferation is followed by epithelialization, angiogenesis, and collagen formation. GT is formed at the end of the proliferation phase. Fibroblasts, collagen, edema, and new blood vessels are formed and undergo maturation in the remodeling phase, resulting in the formation of scar tissue. Collagen is the main protein that contributes to wound strength [8, 39]. The barrier function of the skin is disrupted after thermal injuries, which may result in the development of infections in the wounded area. Infection complicates burn wounds and delays their healing. Therefore, wound dressing should be performed appropriately to prevent the entry of environmental microorganisms [31, 34–40].

We observed that the healing of wounds treated with creams containing 5% and 10%* P. granatum* flower extract was faster than that of wounds treated with the other agents. This may be because of the beneficial effects of* P. granatum* extract on wound healing parameters such as revascularization, fibroplasias, wound contraction, and collagen synthesis. In addition, this beneficial effect of* P. granatum* flower extract may be associated with its antibacterial, anti-inflammatory, and antioxidant properties. These properties of* P. granatum* flower extract probably augment together and promote wound healing compared with the antibacterial effects of standard 1% SSD. Creams containing SSD are commonly used for treating burn injuries because of the antibacterial property of SSD. Although creams containing SSD are recommended as the standard treatment for treating burn wounds, the use of SSD may increase the duration of hospitalization [4, 6, 40]. Because creams containing* P. granatum* flower extract promoted faster wound healing than creams containing 1% SSD, the use of these creams may result in a shorter hospital stay of patients with burn injuries. The treatment of wounds with creams containing the* P. granatum* flower extract resulted in better wound contraction than the treatment of wounds with the other agents. On day 15 of the study, the percentage wound contraction in wounds treated with creams containing the* P. granatum* flower extract was 77-78% compared with 32, 40, and 42% for wounds treated with cream containing 1% SSD, base cream, and normal saline, respectively, indicating that the* P. granatum* flower extract had the best effect on wound healing compared with the other agents.

## 5. Conclusion

The results of this study showed that creams containing the* P. granatum* flower extract remarkably improved the healing of burn wounds compared with creams containing standard 1% SSD, base cream, and normal saline. This was probably related to tannins of the* P. granatum* flower extract. Thus, the results of this study support the use of Golnar (*P. granatum*) flowers for treating burn injuries, as mentioned in Iranian traditional medicine.

## Figures and Tables

**Table 1:** Comparison of the percentage of wound contraction between *Punica granatum* and treated groups^*∗*^.

Day	P 5%	P 10%	SSD 1%	BC	N/S
1	0	0	0	0	0
3	−5.7 ± 5.8	−12.7 ± 6.2	−9.8 ± 7.1	−6.3 ± 5.3	−6.4 ± 5.8
7	15.5 ± 9.6	3.8 ± 5.6	−4.1 ± 6.9	9.3 ± 6.2	16.2 ± 7.3
10	44.9 ± 7.5	33.1 ± 8.5	12.9 ± 5.2	12.5 ± 5.4	28.8 ± 8.2
15	78.3 ± 5.6	76.8 ± 5.4	32.5 ± 5.1	40.3 ± 5.9	41.6 ± 6.3
20	96 ± 4.9	96.5 ± 4.2	60.3 ± 3.2	59.3 ± 4.8	66.7 ± 5.1
25	99.7 ± 0.8	100	82.2 ± 2.1	88.8 ± 4.2	93.7 ± 3.5
30	100	—	93.5 ± 1.3	97 ± 2.8	99.3 ± 2.1
33	—	—	97.5 ± 0.4	98.4 ± 1.9	99.5 ± 1.1

^*∗*^Silver sulfadiazine 1% (SSD 1%), normal saline (N/S), base cream (BC), *Punica granatum* flowers cream 5% (P 5%), and *Punica granatum* flowers cream 10% (P 10%).

**Table 2:** Morphology and histopathology of granulation tissue examined in different groups on the 8th day after burn injury^*∗*^.

Components/groups	SSD 1%	N/S	P 5%	P 10%	BC
Monocytic (macrophage histiocyte infiltration)	2	1	2	2	2
Neovascularization	2	1	2	2	1
Fibroblastic proliferation	2	1	2	2	2
Matrix mucopolysaccharide deposition	2	1	2	2	2
Degree of inflammation	3	1	2	3	3
Extent of bacterial colonization	−2	−3	−1	−1	−1
Degree of granulation tissue formation	2	1	2	2	1
Sum of scores	11	3	11	12	10

^*∗*^Silver sulfadiazine 1% (SSD 1%), normal saline (N/S), base cream (BC), *Punica granatum* flowers cream 5% (P 5%), and *Punica granatum* flowers cream 10% (P 10%). These samples were taken randomly from each group.

**Table 3:** Histopathological examination of dermis on 21 days after burn injury in treated groups^*∗*^.

Groups	Degree of scar formation	Matrix & collagenization organization	Extent of hair follicles	Extent of lymphatic ducts	Degree of innervation	Sum
P 5%	3	3	2	1	0	9
P 10%	3	3	3	1	1	11
SSD 1%	2	2	0	0	0	4
Base C	2	2	0	0	0	4
N/S	2	2	1	1	0	6

^*∗*^Silver sulfadiazine 1% (SSD 1%), normal saline (N/S), base cream (BC), *Punica granatum* flowers cream 5% (P 5%), and *Punica granatum* flowers cream 10% (P 10%). These samples were taken randomly from each group.
